# A comprehensive clinical analysis of the use of percutaneous endoscopic debridement for the treatment of early lumbar epidural abscesses

**DOI:** 10.3389/fsurg.2023.1215240

**Published:** 2023-08-14

**Authors:** Yang Yang, Jianhua Li, Zhengqi Chang

**Affiliations:** Department of Orthopedics, 960th Hospital of PLA, Jinan, China

**Keywords:** lumbar infection, epidural abscess, percutaneous endoscopic debridement, drainage, total laminectomy

## Abstract

**Objective:**

The purpose of this study is to evaluate the safety and efficacy of a percutaneous endoscopic debridement and drainage for lumbar infections with early epidural abscesses.

**Methods:**

Eight cases of early epidural abscess underwent lumbar intervertebral space debridement and drainage by percutaneous endoscopic. Laboratory indicators, pathogenic microorganisms and complications were documented, and the ASIA scores were used to assess preoperative and postoperative neurological function changes. Additionally, the VAS was used to evaluate the therapeutic effect.

**Results:**

The average duration of the drainage tube was 11.25 ± 3.96 days (7–20 days), and the epidural abscess was eliminated after the tube was taken out. Postoperative CRP (14.40 ± 12.50 mg/L) and ESR (48.37 ± 16.05 mm/1 h) were significantly lower than the preoperative CRP (62.5 ± 61.1 mg/L) and ESR (75.30 ± 26.20 mm/1 h). The VAS score after the operation (2.50 ± 0.92 points) was significantly lower than the one before the surgery (8.25 ± 0.83 points). 5 patients experienced lower extremity pain and neurological dysfunction prior to surgery, however, after drainage, the lower extremity pain dissipated and the lower extremity muscle strength improved in one patient. All 8 patients were followed up for a period of (28.13 ± 10.15) months, including 3 patients with spinal segmental instability who had lumbar bone graft and internal fixation for the second stage. At the end of the follow-up, all 8 patients were clinically cured without any progressive nerve injury, paraplegia or recurrence of infection.

**Conclusion:**

Percutaneous Endoscopic Debridement and Drainage is an effective way to drain intraspinal abscesses, thus avoiding any potential progressive harm to the spinal cord.

## 1. Introduction

Epidural abscess is a rare spinal infection that was first mentioned by Giovanni Battista Morgagni in 1,761 (1, 2). The rate of this occurrence among inpatients is usually between 0.2 and 2.0 per 10,000 (3); however, with the advancements in imaging technology, the incidence has risen to 2–8/10,000 (4, 5). It is commonly seen in individuals aged between 30 and 60 and there is a male to female ratio of 1:0.56 (6). This infection is serious and requires prompt surgical treatment to prevent long-term neurological damage. The mortality rate is approximately 3.7%–5% (7).

Surgery is the initial treatment option for epidural abscesses, and it is usually combined with antibiotic therapy. A classic surgical procedure is a total laminectomy, which requires the spinous process and both vertebral lamina to be taken out, as well as a portion of the posterior longitudinal ligament to be cut in order to open the intervertebral space and drain the abscess (8, 9). Surgery, however, can bring about several complications such as spinal instability (10), infection spread, and recurrence (11), and a large number of patients are unable to tolerate the procedure, leaving them with no other treatment options. There is disagreement regarding whether epidural abscesses without neurological symptoms should be surgically treated, yet it is essential to intervene in cases of advanced disease and potential neurological damage. However, conventional surgery may aggravate the condition. It is now necessary to find a suitable and minimally invasive approach to drain the abscess, regulate the infection, and control the spinal cord injury.

Owing to the recent developments in minimal invasive technology, Percutaneous endoscopic decompression has become a helpful supplementary treatment for lumbar diseases, and it is also of great use in lumbar infection cases (12). In the Department of Orthopedics at the 960th Hospital of the PLA, 8 cases of lumbar epidural abscess were documented between November 2017 and September 2021, with 4 males and 4 females. The treatment of choice was a combination of percutaneous endoscopic debridement and drainage (PEDD) with antibiotics, resulting in a definite curative effect.

## 2. Materials and methods

### 2.1. General information

During the period from November 2017 to September 2021, the Department of Orthopedics of the 960th Hospital of the PLA managed 8 cases of lumbar epidural abscess, comprising of 4 males and 4 females, with a mean age of 54.50 ± 15.33 years (ranging from 31 to 80 years). Upon admission, the team conducted routine interrogations and physical examinations, as well as x-ray, CT and MRI scans, to confirm the diagnosis of spinnal infection. Patients who have combined urination and defecation disorders, or who have lower limb major muscle group muscle strength lower than Grade 3, those with systemic infection or septic shock, those with incomplete clinical data, and those whose diagnosis is unclear and lack pathological support, will be excluded from the study. All the patients experienced low back pain, and 6 of them had fever. The lesion segments were identified as L1/2 in 1 case, L2/3 in 2 cases, L4/5 in 3 cases, and L5/S1 in 2 cases. One case of epidural abscess was located in the dorsal dural sac, while the remaining cases were located in the ventral dural sac. Additionally, 5 cases had neurological signs in their lower limbs. The onset of the illness was estimated to be between 14 and 120 days, with an average of 22 ± 34.12 days. One patient had diabetes mellitus, another had primary aldosteronism, two had a history of surgery, one had rheumatoid arthritis and was taking long-term oral steroids, two had been confined to bed for more than one month, and four had no complications. Further information can be found in Tables 1–3.

**Table 1:** Analysis of the clinical characteristics of epidural abscess.

No	Age (years)	Gender	Symptoms	Neurological symptoms in the lower limbs	ASIA score	Diseased time (days)	Position	Complication
1	47	Female	Low back pain, fever	–	5	30	L2/3	–
2	80	Male	Low back pain, fever,walking difficulty	Strength of both lower limbs was grade 4, sense of the right lateral leg decreased	4	30	L4/5	Prolonged recumbency bedsore
3	31	Male	Low back pain, numbness of the right lower limb	Right plantar sense reduced, strength of the plantarflexor muscle was grade 3	4	120	L5/S1	–
4	62	Male	Low back pain, fever	–	5	20	L4/5	Primary aldosteronism
5	50	Female	Low back pain, fever, pain and numbness in both lower limbs	Strength of both lower limbs was grade 3, sense of the left dorsum of the foot decreased	3	21	L2/3	Diabetes mellitus, rheumatoid arthritis, long-term oral immunosuppressant, bedsore, bilateral renal abscess, back abscess after surgery
6	62	Female	Low back pain, fever	Extensor strength of the left side was grade 3	4	45	L5/S1	Hypertension
7	63	Female	Low back pain	–	5	30	L1/2	–
8	41	Male	Low back pain, fever, pain in both lower limbs	Sense in the left medial leg decreased	4	14	L4/5	–

**Table 2:** Laboratory and imaging findings.

NO	Spond-ylitis	Intervertebr-al infection	Paraverteb-ral abscess	Bone destruction	WBC (×10^9^/L)	ESR (mm/L)	CRP (mg/L)	Abscess range	Pathogenic bacterium	Pathology
1	+	+	−	−	6.23	50	20.8	L1–L3 ventral	Tuberculosis	Inflammatory granulomas, caseous necrosis
2	+	+	+	+	5.55	82	195	L3-S1 ventral	MRSA	Acute/chronic inflammatory cell infiltration
3	+	+	+	+	5.49	34	52.7	L2-S1 ventral	Tuberculosis	Caseous necrosis
4	+	+	+	+	5.78	54	16.7	L3-L5 ventral	Klebsiella pneumoniae	Acute/chronic inflammatory cell infiltration
5	+	+	+	+	6.84	104	24.6	T12-L2 ventral	Escherichia coli; e.coli	Inflammatory granulomas, inflammatory cell infiltration, sequestrum
6	+	+	−	+	7.18	101	27.5	L5-S1 Ventral/dorsal	−	Acute/chronic inflammatory cell infiltration
7	+	+	−	−	4.87	97	107	T10-L1 ventral	Brucella	Acute/chronic inflammatory cell infiltration
8	+	+	+	+	8.87	80	56	L3-L5 ventral	Brucella	Acute/chronic inflammatory cell infiltration

**Table 3:** Treatment and prognostic analysis.

NO	Antibiotic	Drainage time (days)	ASIA score after drainage	Healing	Follow-up time (months)	ASIA score at the last follow-up	Subsequent operation
1	Rifampin, isoniazid, ethambutol, pyrazinamide	7	5	yes	53	5	–
2	Vancomycin, Linezolid	12	4	yes	41	5	–
3	Rifampin, isoniazid, ethambutol, pyrazinamide	20	4	yes	45	5	–
4	Vancomycin, Imipenem	14	5	yes	36	5	–
5	Piperacillatazobactam, Levofloxacin	10	4	yes	24	5	Posterior approach, bone graft, pedicle screw fixation
6	Cefuroxime	7	4	yes	15	5	Posterior approach, bone graft, pedicle screw fixation
7	Doxycycline; Rifampicin; Compound sulfamethoxazole tablets	10	5	yes	8	5	–
8	Doxycycline; Rifampicin; Compound sulfamethoxazole tablets	10	4	yes	9	5	Extreme lateral approach, Bone graft fusion and internal fixation

### 2.2. Laboratory and imaging findings

All the patients presented with intervertebral space infection complicated by vertebral end plate osteomyelitis or spondylitis, with five patients exhibiting paravertebral abscess and six patients displaying vertebral bone destruction and three patients demonstrating spinal instability. On admission, there was no marked increase in white blood cell count, however, ESR and CRP were both elevated to varying degrees, with two patients exhibiting a notably high CRP level (>100 mg/L). MRI scans revealed inflammatory changes of the intervertebral disc, edematous adjacent vertebral body, osteomyelitis, paravertebral and epidural abscess formation, as well as diffuse enhancement of the intervertebral disc and vertebral body, and cystic enhancement in the epidural space with an irregular rim.

### 2.3 Surgical methods and treatment outcomes

If there were no systemic infection symptoms and ESR and CRP levels were not increasing, PEDD could be conducted under local anesthesia. During the operation, specimens were taken for bacterial culture, drug sensitivity testing, and pathological examination. Patients were placed in prone or lateral decubitus positions, with a thoraco-iliac pillow and abdomen suspended. The C-arm machine was then positioned and the middle line of the iliac crest and spinous process was marked, determining the puncture point. The spinous process was 8–12 cm away, and 1% lidocaine was used for local infiltration anesthesia. With the help of the C-arm, the needle was punctured through Kambin's safety triangle and into the lesion intervertebral space. Then under endoscopy, a visual circular saw can be used to cut away a section of the lumbar facet joint process from the ventral side. Forceps were used to extract the infected nucleus pulposus and endplate tissue until fresh blood was seen. Subsequently, if the imaging reveals the abscess is on the ventral side of the spinal canal, the endoscope should be inserted into the abscess area. Conversely, if the abscess is on the dorsal side of the spinal canal, the endoscope should be inserted into the ventrolateral side close to the abscess. Additionally, plasma electrode was used to remove the necrotic tissue and abscess wall around the lesion. During the operation, 3,000–6,000 ml of normal saline was administered to continuously flush the abscess cavity, and the tissues procured during the operation were retained for bacterial culture, drug sensitivity, and pathological tissue biopsy. Radiofrequency electrocoagulation was employed to suppress bleeding, a two-lumen drainage tube was affixed, and a dressing was applied to cover the incision.

Pathogenic culture results were positive in 3 out of 4 cases, amounting to a positive rate of 75%. This included 1 case of methicillin-resistant Staphylococcus aureus, 1 case of Escherichia coli, and 1 case of Klebsiella pneumoniae, though a specific infection bacterial culture was not done. Patients diagnosed with Mycobacterium tuberculosis were administered a combination of four anti-tuberculosis drugs, namely isoniazid, rifampicin, ethambutol and pyrazinamide, for a period of 12 to 18 months. For Brucella cases, doxycycline, rifampicin and compound sulfamethoxazole tablets were prescribed for 3–6 months. Three patients with positive culture results were treated with antibiotics that were sensitive to the infection, and one patient with negative culture results was given second-generation cephalosporin. The suggested period of antibiotic use is 4–6 weeks, once ESR and CRP have returned to their normal or steady values (13). The indwelling time of the drainage tube was 11.25 ± 3.96 days (7–20 days). The criteria for the removal of the drainage tube were: (1) the patient's pain had gone and the drainage fluid had become clear; (2) there had been a substantial and constant decrease in the Erythrocyte sedimentation rate and C-reactive protein; (3) the bacteria cultures of the blood and drainage fluid were negative on two consecutive occasions (14); and (4) there was no alteration in the drainage fluid's quantity over the course of three days. After the drainage tube was taken out, lumbar MRI was conducted to assess the alterations of the abscess. The pathological findings were mainly characterized by necrotizing exudation, caseous necrosis, both acute and chronic inflammatory cell infiltration, and micropurulent foci. Two patients underwent posterior bone graft fusion and pedicle screw fixation, and one patient went through extreme lateral approach fusion and internal fixation. The average follow-up period was 28.88 ± 16.15 months (ranging from 9 to 53 months).

The ASIA neurological function score, which ranges from 1 to 5 points, was utilized to evaluate the neurological function at admission. Higher scores signify less damage to the neurological function. This score was also re-evaluated after transforaminal endoscopic drainage and at the last follow-up to assess the effectiveness of the neurological treatment.

#### 2.3.1. Statistical methods

This retrospective case series study utilized SPSS 23.0 statistical software (SPSS Corporation, USA) for statistical analysis. The normal distribution data (WBC count, CRP, ESR, VAS score) were expressed as mean ± SD. To compare preoperative and last follow-up results, a paired t-test was conducted with a two-sided alpha value of 0.05.

## 3. Results

The operation duration was 74.31 ± 11.54 min, spanning from 60 to 125 min. Intraoperative blood loss was 21.25 ± 12.41 ml, varying from 10 to 50 ml. The hospital stay was (51.38 ± 16.68) days, between 25 and 82 days. The drainage period was (11.25 ± 4.23) days, with a span of 7 to 20 days. All patients were monitored for 28.13 ± 10.15 months, with a span of 19–36 months. During the procedure, there were no complications such as nerve damage, paravertebral hematoma, cerebrospinal fluid leakage, or meningitis. Five patients with lower limb nerve symptoms had no worsening injury after the operation, and returned to normal after the follow-up. Furthermore, there were no recurrences or low back pain in any of the patients; all of them were able to return to their day-to-day activities and were ultimately cured.

Eight patients underwent PEDD and post-MRI results revealed that the epidural abscess in the spinal canal had vanished once the drainage tube was removed. The WBC count [(5.84 ± 3.14) × 109/L] was lower than that before the operation [(6.35 ± 1.18) × 109/L], yet the difference was not statistically significant (*P* = 0.144, Table 1). In contrast, the CRP and ESR [(14.40 ± 12.50) mg/L and (48.37 ± 16.05) mm/1 h] were notably reduced compared to the pre-operative levels [(62.5 ± 61.1) mg/L and (75.30 ± 26.20) mm/1 h], and the differences were all statistically significant (all *P* < 0.05, Table 4). Eight patients had normal CRP and ESR levels at their last follow-up. Upon the last follow-up, all of the 8 patients no longer experienced any low back pain. Prior to the operation, their VAS scores for low back pain were 8.25 ± 0.83, which was significantly reduced to 2.50 ± 0.92 at the time of drainage tube removal (*P* < 0.05, Table 1). This marked a significant improvement in the low back pain of the patients.

**Table 4:** Experimental comparison before and after operation (x ± s).

Time	WBC (×10^9^/L)	ESR (mm/1 h)	CRP (mg/L)	VAS score
Pre-operation	6.35 ± 1.18	75.30 ± 26.20	62.5 ± 61.1	8.25 ± 0.83
After drainage	5.84 ± 3.14	48.37 ± 16.05	14.40 ± 12.50	2.50 ± 0.92
T-value	1.000	3.040	2.671	10.565
*P*-value	0.351	0.001	0.001	0.001

Prior to the operation, five cases of lower limb neurological dysfunction were observed, with a decrease in muscle strength and hypoalgesia. After the PEDD, the lower limb pain was alleviated in all five patients, and one patient's muscle strength increased from grade Ⅲ to grade 2163;. Following drainage treatment, no further deterioration of lower limb nerve function was reported among the patients, and no lower limb paraplegia, bowel or urine dysfunction occurred. At the last follow-up, 8 patients had regained normal lower limb function.

### 3.1. Typical case

**Figure 1:**
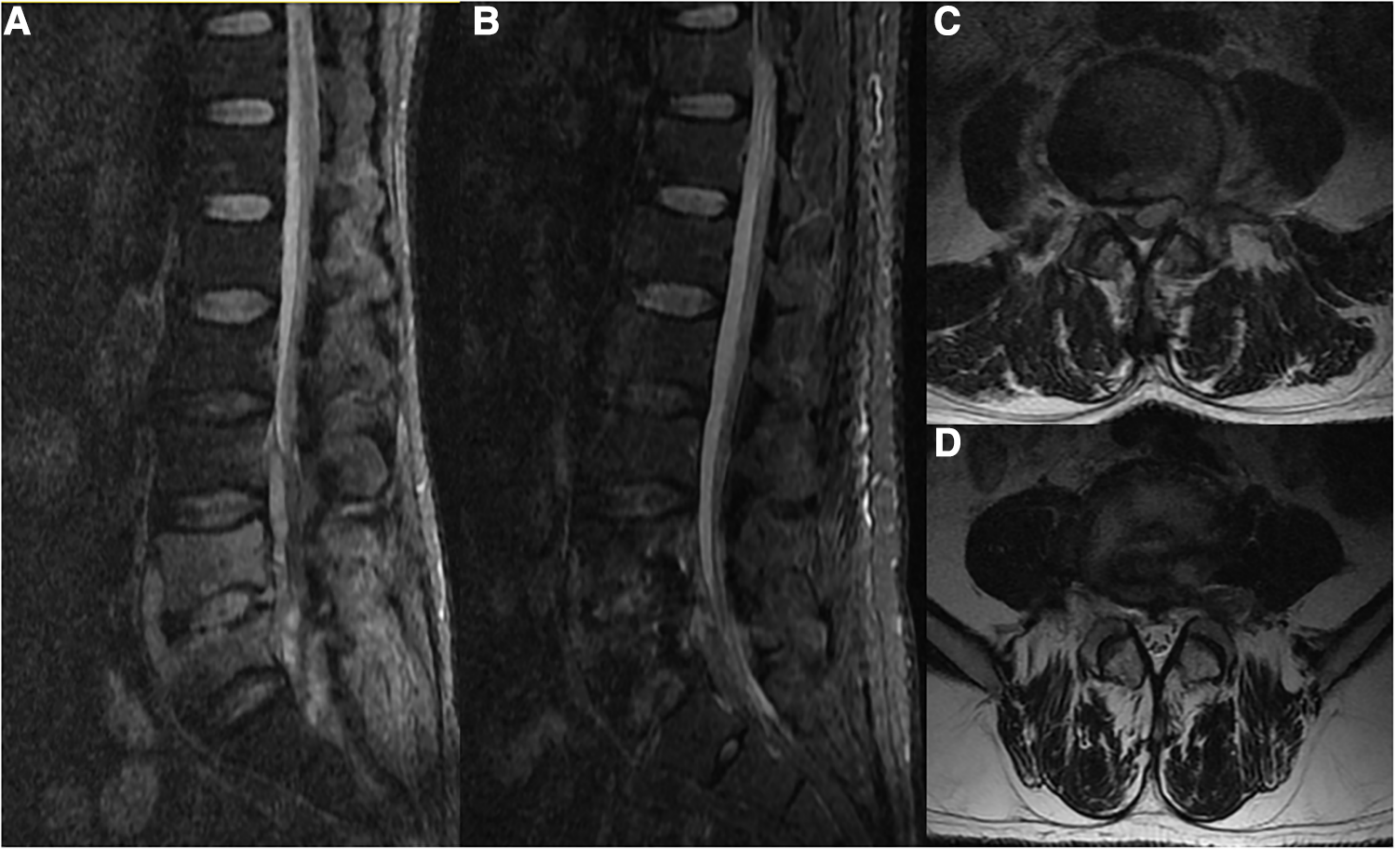
A 40-year-old male presented with Brucella infection of the lumbar spine. (**A,C**) MRI showed low T1 and high and low T2 mixed signals in the L4, L5 vertebral bodies and the L4/5 space, and patchy high and low mixed signals in the spinal canal behind the L5 vertebral body. The space-occupying lesion was located ventral to the left of the spinal cord, which was considered to be a spinal canal abscess. (**B,D**) Reexamination of the lumbar MRI after drainage showed that the signal of the L4 and L5 vertebral bodies was significantly improved, and the space-occupying signal in the spinal canal had disappeared.

A 40-year-old male was admitted to the hospital in November 2021 due to a prolonged history of low back pain with recent radiating pain in both lower limbs for the past 15 days. The patient had also reported irregular fevers with a maximum temperature of 38.3°C prior to admission. On physical examination, the patient exhibited lameness, no deformity of the spine or limbs, tenderness and percussion pain at the level of the L4/5 spinous process radiating to both lower limbs, hypoalgesia in the left anterior knee and medial leg, grade Ⅴ muscle strength in both lower limbs, normal muscle tension in both lower limbs. The left knee tendon reflex is absent, the right knee tendon reflex is present, both Achilles tendon reflexes are present, and there is a lack of patellar and ankle clonus bilaterally. Pathological signs were also negative. Laboratory tests revealed a white blood cell count of 8.87 × 109/L, erythrocyte sedimentation rate of 80 mm/L, and C-reactive protein of 56 hg/L. An x-ray of the lumbar spine revealed degeneration, a slight narrowing of the intervertebral space at L4/5, rupture of the L5 isthmus, and no forward slip of the L5 vertebral body. A CT scan showed hyperplasia of the anterior edge of the L5 vertebral body, “insect erosion-like” bone destruction of the L4 and L5 vertebral bodies, and discontinuity of the L5 bilateral isthmus bone. An MRI revealed low T1 and high T2 mixed signals in the L4, L5 vertebral bodies, and L4/5 space, as well as patchy high and low mixed signals in the spinal canal behind the L5 vertebral body. A space-occupying lesion was located in the ventral spinal cord, which was low signal on T1-weighted image and high signal on T2-weighted image, indicating the presence of a spinal canal abscess (Figure 1). The Brucella agglutination test was positive.

**Figure 2:**
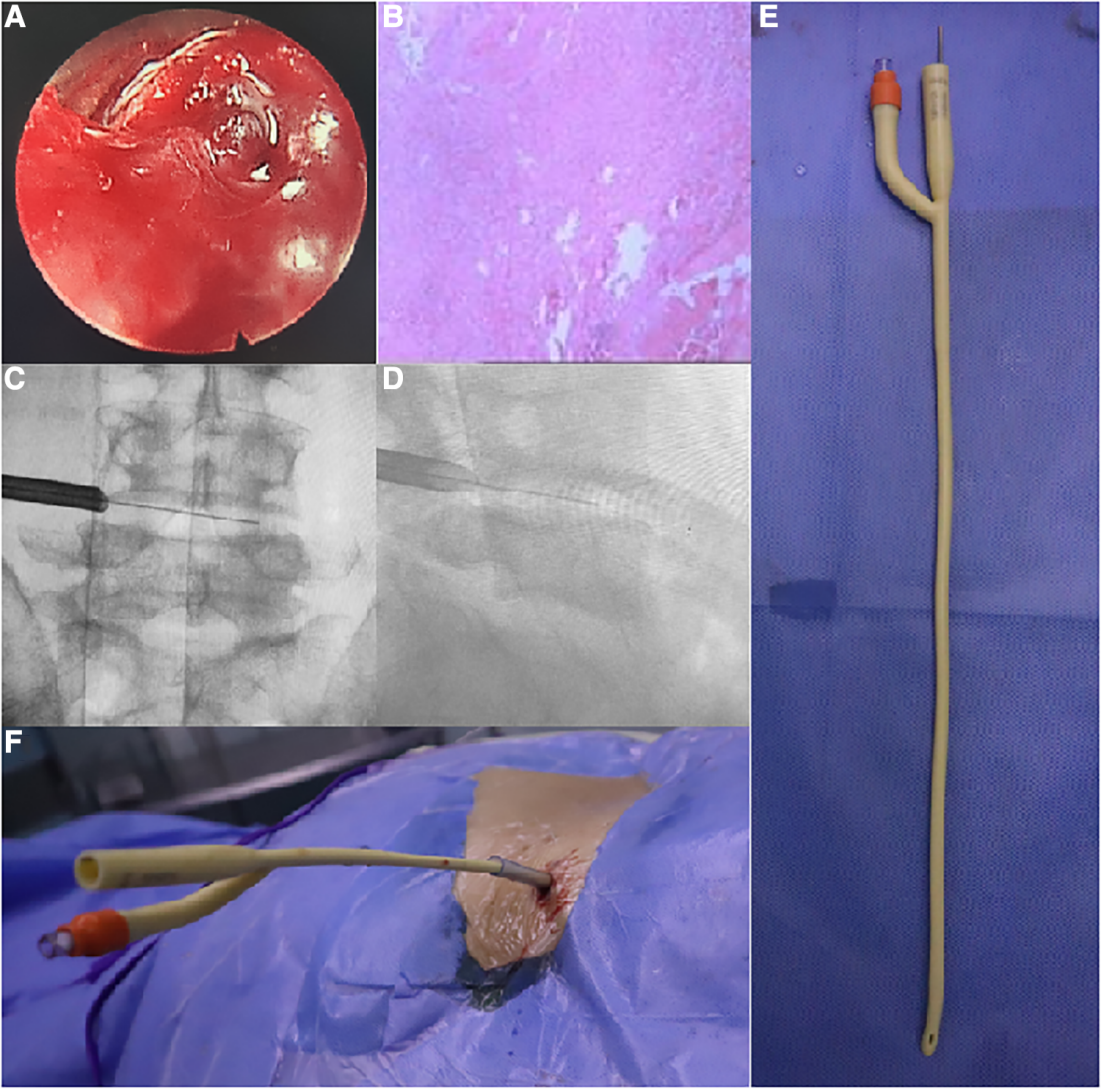
(**A**) Granulation tissue proliferation with inflammatory hemorrhage in the intervertebral space as seen under endoscopy. (**B**) Postoperative pathology showed infiltration and necrosis of a large number of inflammatory cells. (**C,D**) The cannula was located in the intervertebral foramen to open the spinal canal for drainage. (**E,F**) Schematic diagram of the double-lumen drainage tube inserted into the intervertebral space.

Following admission, the patient's general condition was seen to have improved, as well as their nutritional status. PEDD was then performed, with L4/5 foraminoplasty being undertaken (Figure 2). The working channel was inserted into the L4/5 space, which exhibited inflammatory granulation hyperplasia and destruction of the L4 and L5 vertebral bodies. The removed tissue was sent for pathological examination, bacterial culture and drug sensitivity testing. The pathology results revealed the presence of granulation tissue, along with acute and chronic inflammatory cell infiltration. Postoperative drainage was maintained for 9 days, followed by a triple antibiotic regimen of doxycycline (100 mg orally twice a day for 3 months), gentamicin (5 mg/kg intramuscularly once a day for 1 week) and rifampicin (10 mg/kg orally once a day for 3 months) in addition to anti-brucellosis treatment for 3 months. Subsequent lumbar MRI scan revealed the disappearance of the epidural abscess in the spinal canal. The second stage of treatment entailed L4/5 transpolar lateral approach debridement and autogenous iliac bone grafting with internal fixation, performed under general anesthesia. The patient was monitored for 24 months and showed a successful fusion of the L4/5 space, with normal sensory muscle strength in both lower limbs and normal daily activities, with no recurrence.

## 4. Discussion

Epidural abscess is a rare infection of the lumbar spine, with the highest prevalence in the lumbar spine (50%), followed by the thoracic spine (38%) and cervical spine (12%) (15). Clinical manifestations of this condition are usually fever, neck or back pain, and neurological deficits (16), though the specificity of these signs is low (17), with only 8%–15% of patients exhibiting the triad (18). Early diagnosis of epidural abscess is essential, as progressive neurological dysfunction or paraplegia often indicate a poor prognosis (19, 20). Treatment of this condition is based on anti-infective therapy, and the identification of the pathogenic microorganism is critical for the selection of the appropriate drugs (21, 22). In this study, the positive rate of pathogenic bacteria was 75% (3/4). Spinal endoscopic technique can be used to obtain lesion tissue in the early stage, and bacterial culture and drug sensitivity tests can be used to detect pathogenic bacteria and initiate timely and effective anti-infective treatment.

Treatment of epidural abscess is a contentious issue. Retrospective research has suggested the use of antibiotics alone may be effective in some cases (23, 24), however there is a risk of severe complications, such as disease progression and paraplegia, should treatment fail. To date, emergency decompression has been the preferred approach for spinal epidural abscesses, as has been demonstrated in multiple studies (25, 26). However, traditional laminectomy decompression and drainage carries several issues, such as large surgical trauma, inability to stabilize the spine in one operation, and difficulty in managing infected incisions post-surgery. Furthermore, it can be difficult to determine the right time for surgery, as the progress of the disease is often sudden and without warning signs. Additionally, some patients may not be able to undergo traditional general anaesthesia due to underlying conditions.

The use of minimally invasive technology has enabled researchers to utilize percutaneous endoscopic drainage (PEDD) to treat lumbar infections (27, 28). Omar et al. (29) first reported the successful use of PEDD to treat a patient with spinal canal abscess, and the results were satisfactory. Eight patients with epidural abscesses underwent PEDD, which was well tolerated under local anesthesia. During the procedure, the infected intervertebral space tissue was initially debrided, and the articular process was formed to facilitate drainage. The operation time was short (74.31 ± 11.54 min), and the intraoperative blood loss was minimal (21.25 ± 12.41 ml), significantly lower than reported in the literature (30). After drainage, the inflammatory markers such as ESR and CRP were significantly decreased compared to pre-operation values, which was statistically significant (*P* < 0.05 Table 4); the VAS scores before and after the operation were also significantly reduced, and the difference was statistically significant (*P* < 0.05 Table 4). This was likely due to the decrease in intervertebral space pressure after drainage, allowing the epidural abscess to flow back into the intervertebral space and be drained from the body. MRI scans following the removal of the drainage tube in 8 patients showed that the abscess in the spinal canal had disappeared, indicating that the clinical treatment was effective.

Early detection and prompt intervention of epidural abscess is of utmost importance for the successful management of the condition. Neurological function evaluation is a key factor in determining the severity of spinal cord damage. In this study, 5 of the 8 patients underwent preoperative nerve injury and were classified as ASIA grade 3–4. After treatment with PEDD, these patients reported a significant reduction in lower limb pain symptoms, likely due to the decreased pressure and inflammatory stimulation to the dura and nerve root. Moreover, the VAS score was found to have decreased significantly postoperatively, thus indicating the effectiveness of PEDD in preventing irreversible nerve damage caused by epidural abscess. One patient even recovered from a grade 3 to a grade 4 ASIA score. Therefore, PEDD is an important tool in treating early epidural abscess and its associated neurological symptoms.

In this study, eight patients underwent successful drainage tube removal, with no further infection spread. MRI scans revealed that the epidural abscess had disappeared in (11.25 ± 4.23) days, which was notably shorter than the expected 4–8 weeks of conservative treatment with antibiotics alone, reported in the literature (31). Subsequent treatments included vertebral destruction and spinal stabilization in three patients, and secondary consecutive screw internal fixation combined with autologous iliac bone graft fusion in two patients, and one patient had lumbar internal fixation with autologous iliac bone graft fusion via an extreme lateral approach. The bone graft fusion was successful at the final follow up, without recurrence of the infection. These three patients underwent debridement and fusion of the intervertebral focus, with the first intervertebral foramen endoscopic surgery leading to improved results and a decrease in the size of the second operation. This study suggests that PEDD technology combined with antibiotics is an effective method of controlling the spread of infection, and could have positive effects on the ultimate treatment.

In conclusion, Percutaneous Endoscopic Drainage (PEDD) has several advantages in the treatment of an epidural abscess. These include the ability to obtain early pathogenic tissue to guide antibiotic selection, the use of local anesthesia with a short operation time and minimal injury, the expansion of surgical indications, the reduction of abscess disappearance time, and the prevention of irreversible nerve injury. However, it should be noted that this study was limited by its small sample size and retrospective nature, and further research is needed.

## Data Availability

The original contributions presented in the study are included in the article/Supplementary Material, further inquiries can be directed to the corresponding author.
