# Supplementary material for: A comprehensive clinical analysis of the use of percutaneous endoscopic debridement for the treatment of early lumbar epidural abscesses
Source: Front Surg. 2023 Aug 14;10:1215240. doi: 10.3389/fsurg.2023.1215240 (PMC10461046; doi:10.3389/fsurg.2023.1215240)
Supplement: Supplementary file 1 [file Table1.docx]

*Preoperative and postoperative imaging data of 3 patients*

Case1


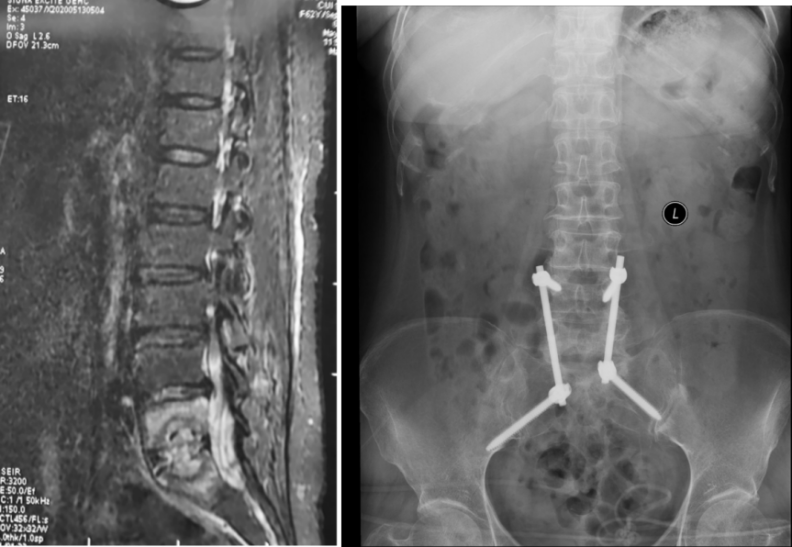


Case 2


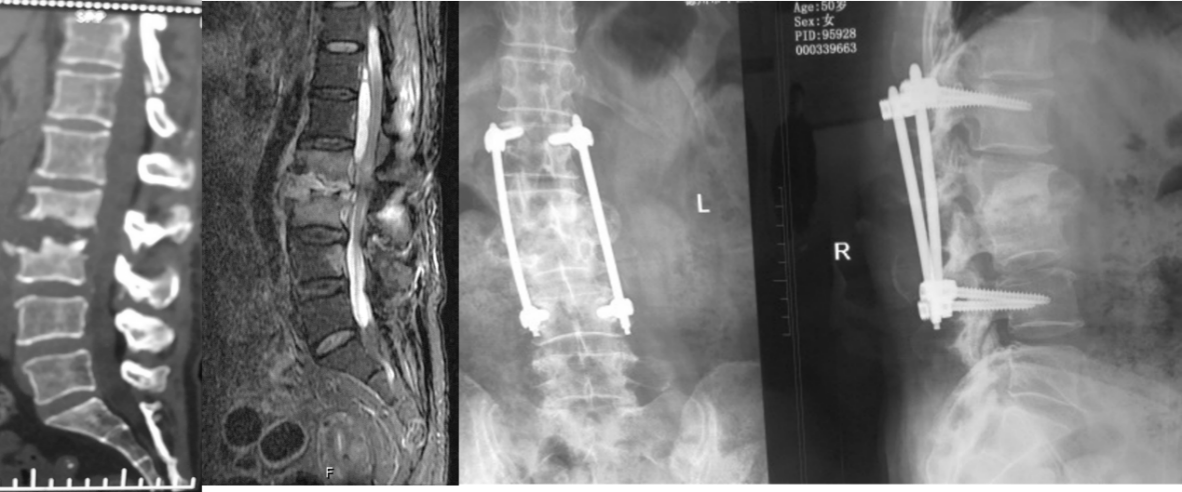


Case 3


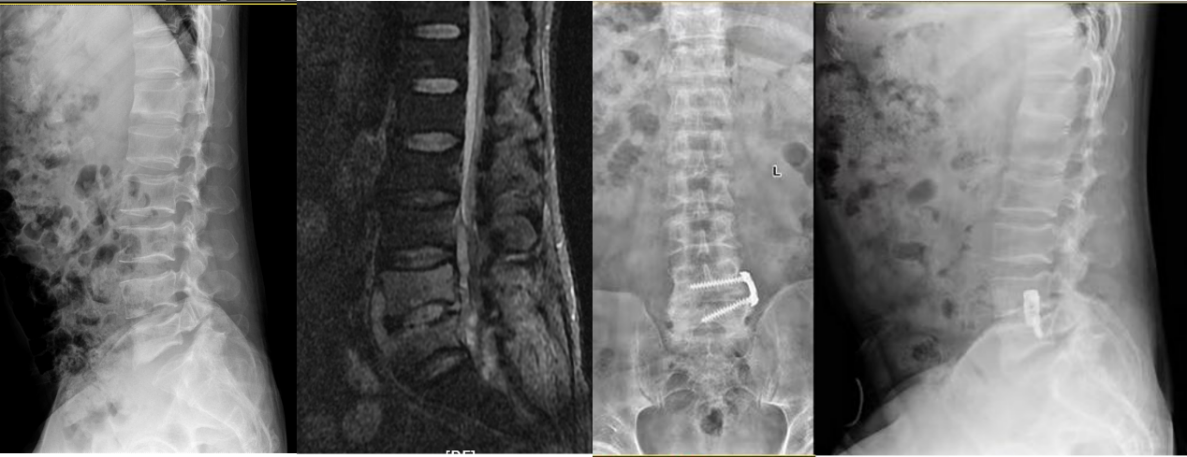


1. There is a mention of a microscope on page 9, but I assume that was a mistake and will be corrected. Overall English can be improved.

*Respond: The reviewer's comments are correct, and we have revised them.*
